# Does health securitization affect the role of global surgery?

**DOI:** 10.1007/s10389-020-01347-3

**Published:** 2020-07-30

**Authors:** Isabella B. Metelmann, Steffen Flessa, Alexandra Busemann

**Affiliations:** 1 Department of Visceral, Transplant, Thoracic and Vascular Surgery, University Hospital of Leipzig, Liebigstrasse 20, 04103 Leipzig, Germany; 2 Institute of Health Care Management, University of Greifswald, Friedrich-Loeffler-Str. 70, 17489 Greifswald, Germany; 3 Department of General, Visceral, Thoracic and Vascular Surgery, Universitätsmedizin, University of Greifswald, Ferdinand Sauerbruchstr, 17457 Greifswald, Germany

**Keywords:** Global surgery, Securitization, Infectious diseases, Health security

## Abstract

**Aim:**

More and more frequently outbreaks of infectious diseases force the international community to urgent health action and lead to an increasing security focus on global health. Considering the limiting character of resource allocation, all other medical conditions must compete with the top spot of health security matters, as we currently see with the outbreak of COVID-19. Surgery is an integral part of universal health offering life-saving therapy for a variety of illnesses. Amidst the increasing nexus of infectious diseases and health security and in the view of *Public Health Emergencies of International Concern* (PHEIC), is there a risk of global surgery falling behind?

**Subject and Methods:**

While the global undersupply of surgical care is well recorded, contextual explanations are absent. Our research introduces the constructivist concept of securitization according to the Copenhagen School to explain the structural handicap of global surgery and by that presents a structural explanation. We investigate the securitizing potential of surgical diseases in comparison to infectious diseases.

**Results:**

Surgical conditions are non-contagious without the risk for disease outbreaks, hardly preventable and their treatment is often infrastructurally demanding. These key features mark their low securitizing potential. Additionally, as *PHEIC* is the only securitizing institution in the realm of health, infectious diseases have a privileged role in health security.

**Conclusion:**

Surgery substantially lacks securitizing potential in comparison to communicable diseases and by that is structurally given an inferior position in a securitized health order.

## Introduction

More and more frequently, infectious diseases throw the world into a dither. Fighting pandemic spread of SARS COV-2 for example has become an international matter of security policy. The situation was called a war by The New York Times’ headline Feb. 28, 2020: “In China’s War on the Coronavirus, a community is besieged”, and the national leader, president Xi Jingping assures that the Communist Party is “a robust shield providing holistic security” (Qin and Wee 2020). Does this health-centred security policy include global surgery as well? Amidst the increasing nexus of infectious diseases and health security and in the view of the recently declared *Public Health Emergency of International Concern* (PHEIC), is there a risk of non-communicable falling behind?

The Sustainable Development Goals (SDG) marked “Good health and wellbeing” as a substantial part of human development incorporating the whole range of medicine. Surgery is an integral part of universal health offering life-saving therapy for a variety of illnesses. Surgery encompasses a wide-reaching spectrum of diverse treatments ranging from the incision of cutaneous abscess to complex procedures such as heart transplant or elective interventions such as most plastic and reconstructive surgeries. While all these operations are favourable in highly developed health systems, low- and middle-income countries (LMIC) often lack even the simplest ones. Data show, that global surgery is significantly underrepresented in our modern world: worldwide, 5 billion people do not have access to safe and timely surgery (Meara et al. 2015). These people mostly live in LMIC typifying a substantial mismatch in the global distribution of surgical care (Meara et al. 2015; Weiser et al. 2008). In 2010, untreated surgical emergency conditions led to nearly 900,000 deaths, meant 20 million years of life lost (YLL) and 25 million disability-adjusted life years (DALYs) (Stewart et al. 2014). Annually, 81 million people are at risk of catastrophic expenditure when they seek surgical care (Shrime et al. 2015). From a macroeconomic perspective, the burden of untreated surgical conditions leads to significant losses in GDP, projected to be 2.5% in Sub-Saharan Africa in 2030 (Alkire et al. 2015). In the light of limited resources, it is obvious that in the sense of universal health coverage and cost-effectiveness, the spectrum of necessary interventions needs to prioritize emergency and essential surgical care. There is no given consensus on how to define basic surgery, and it is obvious that this is highly influenced by local circumstances. Nonetheless, some easy and cost-effective procedures are broadly acknowledged as basic surgical techniques that should be available in all settings, such as abscess and wound management, caesarean section, appendectomy, hernia repair, laparotomy, tracheostomy, cleft lip repair, clubfoot repair, fracture treatment and cataract surgery (World Health Organization 2007; Bickler et al. 2015; Higashi et al. 2015). These procedures have proven that their implementation in LMIC is cost-effective (Gosselin et al. 2006; Gosselin and Heitto 2008; Chao et al. 2014; Grimes et al. 2014).

Content-related reasons for the global underrepresentation of surgery and its role as “the neglected stepchild of global health” (Farmer and Kim 2008) are not well known. However, considering the limiting character of resource allocation, surgery must compete with the top spot of health security matters.

In the 1980s, the *Copenhagen School* around Barry Buzan, Ole Wæver and Jaap de Wilde developed the concept of *securitization*, (Buzan and Hansen 2009; Buzan 1983, 1995; Buzan and Wæver 2003; Buzan et al. 2013; Wæver 1995). Named by Bill McSweeney (McSweeney 1996), the Copenhagen School is a group of European scholars with Barry Buzan and Ole Wæver as its most influential and prominent representatives. Having published a number of significant works in the field of security studies since the early 1980s (Buzan 1983; Buzan et al. 2013; Buzan 1995; Buzan and Wæver 2003; Wæver 1995), they then focused on the concepts of societal security and securitization (Buzan and Hansen 2009). Securitization in the sense of the Copenhagen School referred to the purposeful identification of a potential existential threat that arises from a specific issue with the intention to receive immediate and priority action, which allows measures to be taken that would not be justified under regular conditions (Buzan et al. 2013). Thereby, theoretically any public issue can be securitized (Buzan et al. 2013). The securitization of highly contagious infectious diseases is a textbook example for securitization of health (Wishnick 2010; Lo Yuk-ping and Thomas 2010; Caballero-Anthony 2006). According to Dannreuther, the concept of securitization is “the most influential attempt to develop a middle-range theory for security studies, incorporating the methods and methodology of constructivism” (Dannreuther 2013).

The aim of our research is to discuss the structural handicap of global primary surgery in comparison to infectious diseases due to the increasing importance of global health security considering the securitization in the sense of the Copenhagen School.

## The evolution of global health security

Global health security is a term which has garnered significant scientific attention and controversy, having profited from the intellectual transition of security concepts during the past 15 years, as the nation-centred focus has been broadened to also encompassing individual and community security threats (Takemi et al. 2008). For a long time, health and security were mainly seen as unidirectionally linked, in the sense that conflict directly and indirectly causes health problems (McInnes 2008). Yet, when trade begun to grow to an international scale in the nineteenth century, the risk of foreign infectious diseases coming to Europe became prevalent, and internationally agreed health regulations became meaningful (McInnes 2008). This perception diminished in the late 1960s, when medical developments opened up possibilities to save a growing number of lives, and the introduction of antibiotic therapy seemed to have solved the burden of infectious diseases (Fauci 2001). However, the emergence of new diseases (e.g. HIV/AIDS) or re-emergence of diseases that were previously thought vanquished (e.g. dengue and West Nile fever) brought communicable diseases to new prominence (Fauci 2001). By the end of the Cold War, the changing political world order and increasing globalization also brought new actors to the stage, who were seen to pose a risk to international peace and stability. Scientific progress and global cross linking brought bioterrorism to new significance.

There are two main factors associated with the rising significance of health as a security issue in the late 1990s: the first is a shift from military-focused security studies to a broader science after the end of the Cold War, allowing a formerly “soft science” to get on the security agenda, such as environment and health. The second is simply human agency, with advocates making significant efforts to bring attention to health security issues. Gro Harlem Brundtland, former head of WHO, placed a strong emphasis on health security, stating that “we must broaden the debate, to accept that health is an underlying determinant of development, global security and stability” (Brundtland 2003). By that time she had already acknowledged the jeopardizing potential Ebola might have in unstable areas (Brundtland 2003).

A second influential advocate for bringing health security to international attention was former US President Clinton’s ambassador to the UN, Richard Holbrooke, who made a significant endeavour to get HIV/AIDS onto the UN SC’s agenda after returning from a visit to southern Africa in 1999 that left him with a lasting impression of the burden of the disease on the African people (BARNETT and PRINS 2006). However, scholars are unclear whether Holbrooke’s motivation was in fact security concerns, or whether he was trying to increase its political potential (McInnes 2008). Regardless, HIV/AIDS became an issue on the UN SC’s agenda, and was considered in the first ever health-related UN SC resolution, No. 1308, which stressed “that the HIV/AIDS pandemic, if unchecked, may pose a risk to stability and security” (United Nations Security Council 2000). HIV/AIDS was declared to pose an international threat for four main reasons: (1) the economic burden arising from expensive treatment and loss of work for infected people in the most productive period of their lives (International Crisis Group 2001), (2) the high infection rates among military personnel, leading to a decline in countries’ military capacity (Feldbaum et al. 2006), (3) the risk of international spread of HIV/AIDS by peacekeeping forces, as they are exposed to high HIV prevalence rates in the top 10 contributing nations to peacekeeping operations (Feldbaum et al. 2006; UNAIDS 2003), and (4) the strong link between HIV/AIDS and conflict settings, where it can be spread as a means of war by sexual violence (International Crisis Group 2001) or through migration, and preventive measures are even more difficult to set up in unstable areas.

However, these risks are broadly questioned, since empirical evidence is scarce (McInnes and Rushton 2013). In contrast, scholars found evidence the impact HIV/AIDS can have on armies’ capacities was overestimated and simultaneously showed that risk assumptions for the impact high prevalence rates can have on state stability were not evidence-based (Whiteside et al. 2006). Nonetheless, scholars acknowledge that absence of proof does not mean that HIV/AIDS poses no danger to state security (Garrett 2005). Either way, the acknowledgement by the UN SC that an infectious disease could be a threat to international security marked a turning point in global health politics (Elbe 2011).

Another crucial turning point was the 1994 UN Human Development Report (UNHDR), when the UN understanding of security shifted markedly from state security to human security. Human security is described as having four main attributes: it is universal, interdependent, people-centred and easier to achieve through prevention than later intervention (United Nations Development Programme 1994). Henceforth, the widened concept of security included economic, food, health, environmental, personal, community and political security (United Nations Development Programme 1994). “Disease” in this context means a specifically identified threat to human security.

## Who controls the agenda? The prioritization of securitized health matters

On the basis of the assumption that western HIC dominate the scientific discussion and global health agenda setting, van der Rijt and Pang investigated the share western scientists, institutions and journals take in global health and proved that global health programmes and frameworks are designed by people who are not affected by them (van der Rijt and Pang 2013). Shawar et al. came to a similar result seeing that more than two thirds of the participants in expert interviews live in HIC (Shawar et al. 2015).

In LMIC, planning and financing of health services is broadly influenced by international organizations, bilateral aid organizations or NGO (Harman 2014). When *Millennium Development Goals* came into force, African healthcare systems shifted from basic health care to a focus on measures against HIV/AIDS, malaria and to improve child and maternal health (Harman 2014). The influence of external donors and NGO grew, and vertical programmes came into new prominence as their success is easily measurable and quantifiable. Subsequently, the spectrum of health care providers widened causing a parallel system to the public health sector (Harman 2014). The variety of stakeholders in global health governance lead to competition among the actors making exactly those programmes attractive that are short-term effective, media-effective, generate large donations and are prestigious. This applies particularly to western securitized health matters.

Expenditure tracking is a key prerequisite for efficient funding of global health. While development assistance for surgery is thought to be minimal, robust data on spending is missing (Dieleman et al. 2015b). This simultaneously hinders revealing gaps in financing and strengthening surgery’s role. Development assistance for health (DAH) grew substantially during the past thirty years in all health areas (Dieleman et al. 2014; Dieleman et al. 2015a). However, its distribution still shows relevant inefficiencies and is insufficiently structured along countries’ needs (Schieber et al. 2007). DAH databases do not disaggregate data for surgery specifically. However, when referred to its global burden even NCD in total remain alarmingly underrepresented (Chang et al. 2019). While accounting for nearly 50% of disease burden, NCD received only 1.5% of DAH (Dieleman et al. 2014). In contrast, HIV/AIDS earns nearly half of DAH accounting for 3.7% of disease burden (Dieleman et al. 2014). In 2013, low-income countries with a high burden of HIV/AIDS earned 200 times more disease-specific spending than a low-income country with a high burden of non-communicable diseases (Dieleman et al. 2014). The pattern that funding aligns insufficiently with the actual burden of diseases continues in surgery as well: Gutnik et al. showed in two different studies, that financial contributions to global surgery favour vertical programmes in elective surgery, namely ophthalmologic and cleft/lip palate surgery and underrepresents emergency care and local capacity building such as training programmes (Gutnik et al. 2015; Gutnik et al. 2016).

## Discussion

Infectious diseases have an outstanding potential for securitization. Besides a rapid dissemination of diseases, they are often targeted in international campaigns due to easy establishment and manageability of prevention programmes. Programmes for awareness, vaccination or infection protection can be run without doctors or even by non-medical personal, if the procedure is established. Often, spread of disease can be hindered by basic sanitation alone. All these characteristics do not fit for surgical diseases. There is no broad mobilization of people for reasons of fear of infection. Surgical diseases are not likely to spread globally and therefore lack the potential to motivate prompt change of political agenda. Prevalence of surgical conditions remains stable. There are no outbreaks that attract new political attention. As for all non-communicable diseases, there are no short-term and resource-effective prevention measures. A worldwide vaccination campaign against polio is not effective to reduce the number of casualties. Travel restrictions may be effective to contain Ebola or Corona to some extent, but they are apparently not useful in the prevention of appendicitis. Sanitation programmes can limit the spread of cholera but is not useful to avoid hernia. Containing infectious diseases often asks for a comprehensive interaction of logistic and infrastructural expertise, that is not only of medical nature. These chances for delegation are scarce in surgical procedures. Surgical therapies are frequently highly complex and infrastructurally demanding in a personnel, material and procedural matter.

Buzan et al. consider that institutionalization of securitization may arise when there is a persistent threat requiring permanent attention and “constant drama does not have to present, because it is implicitly assumed that when we talk of this (...), we are by definition in the area of urgency” (Buzan et al. 2013). In the realm of health, a *Public Health Emergency of International Concern* (PHEIC) can serve as a health security institution. The PHEIC is the instrument of the 2005 revised International Heath Regulations (IHR) and can be declared by the WHO Secretary General (World Health Assembly 2008). PHEIC “means an extraordinary event which is determined (…) (i) to constitute a public health risk to other States through the international spread of disease and (ii) to potentially require a coordinated international response” (World Health Assembly 2008). In doing so, the Secretary General is authorized to give temporary recommendations, which are non-binding, disease-specific advice for the duration of the PHEIC that supplement standing recommendations, i.e. risk-specific advice for routine or periodic application during ongoing health threats (World Health Assembly 2008). These recommendations mainly focus on trade and travel, aiming to contain international spread of the disease while simultaneously minimizing disruption to international traffic (World Health Assembly 2008). In its origin, the IHR included control measures for specific diseases (plague, cholera and yellow fever) (World Health Assembly 1983). One of the innovations in 2005 was that IHR no longer specify particular diseases or transmission methods, but cover all medical conditions that may impose an international risk (World Health Assembly 2008). To date, WHO has declared five PHEIC: H1N1 influenza in 2009, polio in 2014, Ebola in 2014, Zika in 2016 and recently COVID-19 in 2020. Even though PHEIC is not reserved for specific conditions, it is obvious that it is only applicable for communicable diseases given the need for an international spread of disease. This elevates PHEIC to a securitizing institution for communicable diseases.

Beyond that, global surgery lacks an influential lobby. According to expert interviews unifying stakeholders that force international attention are absent (Shawar et al. 2015). Reasons for that might be disagreement among surgical leaders and the missing of relevant windows of opportunity such as the *Millennium Development Goals (MDG)* (Shawar et al. 2015). With the closing MDG, Brigit Huber published an article with the connotating title “Finding surgery’s place on the global health agenda” and shed light on the necessity to recognize surgery as an integral part on the international health agenda (Huber 2015). Farmer and Kim highlighted in 2008 that in contrast to, for example, HIV/AIDS or tuberculosis, there was not even a Global Fund for Surgery to ensure stable funding (Farmer and Kim 2008). Intensified efforts of global surgery leaders led to a fruitful cooperation with the World Bank Group giving support in data collection and advocacy (Peters et al. 2019). Still, the financial flows for global surgery remain non-transparent with a multitude of actors involved and a missing specification of donations for surgical matters.

The Lancet Commission for Global Surgery laid the foundation for reliable data on global surgical supply and opened a window of opportunity for international recognition and campaigning. The ongoing task to ensure a safe and affordable access to surgery globally is to keep this window open even in times of war against epidemic diseases.

## Summary

Numerous studies have shown an alarming undersupply of basic surgical care in LMIC. Reasons for that remain largely unexplored. We present a new theoretical approach by applying the concept of securitization to the global health agenda arguing that the low securitizing potential of surgical diseases is one reason for its underrepresentation in global health.
